# Post‐activation potentiation after isometric contractions is strongly related to contraction intensity despite the similar torque–time integral

**DOI:** 10.1113/EP091700

**Published:** 2024-04-10

**Authors:** Pauline Eon, Marc Jubeau, Thomas Cattagni

**Affiliations:** ^1^ Nantes Université, Movement ‐ Interactions ‐ Performance, MIP, UR 4334 Nantes France; ^2^ Laboratory Culture Sport Health Society (UR 4660), Sport and Performance Department UFR STAPS, University of Bourgogne Franche‐Comté Besançon France

**Keywords:** electrical stimulation, knee extensors, muscle strength, neuromuscular performance, quadriceps, twitch

## Abstract

Post‐activation potentiation (PAP) is defined as an enhanced contractile response of a muscle following its own contractile activity and is influenced by the intensity and duration of the conditioning contraction. The aim of this study was to determine if the combination of intensity and duration, that is, torque–time integral (TTI) is a determinant of PAP amplitude. We compared PAP amplitude following low‐to‐maximal voluntary conditioning contraction intensities with and without similar TTI in the knee extensors. Twelve healthy males completed two experimental sessions. Femoral nerve stimulation was applied to evoke single twitches on the relaxed quadriceps before and after isometric conditioning contractions of knee extensors. In one session, participants performed conditioning contractions without similar TTI (6 s at 100, 80, 60, 40 and 20% maximal voluntary contraction (MVC)), while they performed conditioning contractions with similar TTI in the other session (6 s at 100%, 7.5 s at 80%, 10 s at 60%, 15 s at 40%, and 30 s at 20% MVC). In both sessions, PAP amplitude was related to conditioning contraction intensity. The higher the conditioning contraction intensity with or without similar TTI, the higher PAP. Significant correlations were found (i) between PAP and conditioning contraction intensity with (*r* ^2^ = 0.70; *P *< 0.001) or without similar TTI (*r* ^2^ = 0.64; *P *< 0.001), and (ii) between PAP with and without similar TTI (*r* ^2^ = 0.82; *P *< 0.001). The results provide evidence that TTI has a minor influence on PAP in the knee extensors. This suggests that to optimize the effect of PAP, it is more relevant to control the intensity of the contraction rather than the TTI.

## Abstract (highlights)

- **What is the central question of this study?**
  Post‐activation potentiation is defined as an enhanced contractile response of a muscle following its own contractile activity and is greatly influenced by the intensity and duration of the conditioning contraction: is the combination of intensity and duration, that is, the torque–time integral, a determinant of post‐activation potentiation amplitude?
- **What is the main finding and its importance?**
  The main findings of this study provide evidence that the torque–time integral has a minor influence on post‐activation potentiation in the knee extensors. This suggests that to optimize the effect of post‐activation potentiation, it is more relevant to control the intensity of the contraction rather than the torque–time integral.

## 1 INTRODUCTION

The contractile response of a human skeletal muscle is influenced by its contractile history. In contrast to fatigue, which decreases the contractile performance of a muscle (Gandevia, 1992), post‐activation potentiation (PAP) is a mechanism that enhances muscle contractile performance (Brown & Euler, 1938; Krarup, 1981; Sale, 2004) and sport movements (Boullosa et al., 2018; Garbisu‐Hualde & Santos‐Concejero, 2021; Seitz & Haff, 2016). Thus, PAP is receiving great interest from sport science researchers, coaches and athletes.

PAP is defined as an enhanced contractile response of a muscle following its own contractile activity (Brown & Euler, 1938; Krarup, 1981). It occurs in response to phosphorylation of regulatory myosin light chains allowing movement of the myosin head to the actin filament and increasing the rate of the cross‐bridge cycle (Greenberg et al., 2009; Uyeda et al., 1996). It is commonly assessed in vivo from the maximal twitch force amplitude evoked by a supra‐maximal electrical stimulation (Gago et al., 2017; Jubeau et al., 2010; Prieske et al., 2018; Vandervoort et al., 1983). The increase in twitch force amplitude between before and after a muscle contraction indicates the amplitude of PAP.

The magnitude of PAP can be influenced by several conditioning contraction characteristics such as contraction intensity and duration (Vandervoort et al., 1983). Previous findings evidenced a positive relationship between the magnitude of PAP and the conditioning contraction intensity: the higher the conditioning contraction intensity, the higher the PAP (Fukutani et al., 2014; Mettler & Griffin, 2012; Sasaki et al., 2012; Vandervoort et al., 1983). For instance, Sasaki et al. (2012) reported that the magnitude of PAP increases with contraction intensity from 30 to 100% maximal voluntary contraction (MVC) of plantar flexors. The greater PAP observed after high‐intensity contraction can be explained by the recruitment of fast‐twitch fibres (Type II) (Brown & Loeb, 1998; Grange et al., 1993; Hamada et al., 2003; Houston et al., 1985; Moore & Stull, 1984; Vandervoort et al., 1983), which have a greater capacity for myosin light chain phosphorylation in response to high‐frequency activation (Grange et al., 1998; Sweeney et al., 1993). Previous findings also evidenced that the duration of conditioning contraction is an important factor that accounts for the variability of PAP (Manning & Stull, 1982; Vandervoort et al., 1983). For instance, PAP appeared to be optimized after a 10‐s MVC compared to 3 or 30 s (Vandervoort et al., 1983). It tends to reach a plateau or slightly decrease from 10 to 30 s of MVC, and greatly decrease for MVC longer than 30 s (Vandervoort et al., 1983) because of fatigue (Behm et al., 2004; Rassier & MacIntosh, 2000). At submaximal contraction intensities, Mettler and Griffin (2012) demonstrated that the conditioning contraction duration needed to achieve maximal PAP decreases as conditioning contraction intensity increases.

Since both the intensity and duration of the conditioning contraction have a complementary impact on PAP, the torque (or force)–time integral (TTI or FTI), that is, the intensity × duration combination of isometric conditioning contraction, has been proposed as a key determinant of the magnitude of PAP (Mettler & Griffin, 2012). Mettler and Griffin (2010) compared the PAP on adductor pollicis muscle through electrically evoked conditioning contractions with different frequencies and pulses number without similar FTI. First, a positive correlation between FTI and the magnitude of PAP (*r* = 0.7) was found, highlighting that PAP is moderately influenced by FTI. Second, the increase of PAP with the increase in voluntary conditioning contraction intensity without similar FTI was confirmed (Mettler & Griffin, 2012). However, these authors found that PAP is no longer influenced by the intensity of conditioning contraction when FTI is similar to the conditioning contractions (Mettler & Griffin, 2012). Despite this result, the method used in the studies of Mettler & Griffin (2012, 2010) cannot support the conclusion of no effect of conditioning contraction intensity on PAP when FTI is similar in human muscle. Indeed, in their first study (Mettler & Griffin, 2010), the authors used evoked conditioning contractions while PAP is lower for evoked than voluntary conditioning contractions (Jubeau et al., 2010). In both studies, they assessed PAP on the adductor pollicis muscle, which contains a high level of slow‐twitch fibres (about 80%) (Vikne et al., 2012), which are poorly sensitive to PAP compared with fast‐twitch fibres (Brown & Loeb, 1998; Grange et al., 1993; Hamada et al., 2003; Houston et al., 1985; Moore & Stull, 1984; Vandervoort et al., 1983). Finally, in the second study (Mettler & Griffin, 2012), they used low levels of conditioning contractions (25 and 50% MVC), which were probably not able to recruit fast‐twitch fibres and thus to induce a high level of PAP (Fukutani et al., 2014; Hamada et al., 2003; Sasaki et al., 2012; Vandervoort et al., 1983). To better understand the effect of conditioning contraction intensity with similar FTI or TTI, it is necessary to investigate PAP with voluntary conditioning contractions at both low and high intensities and on muscles with higher levels of fast‐twitch fibres such as quadriceps muscles.

In this context, the purpose of the present study was to compare PAP following low‐to‐maximal voluntary conditioning contraction intensities with and without similar TTI in the knee extensors. Since we assumed that conditioning contraction intensity is the main determinant of PAP, we hypothesized that PAP would be greater at high than at low levels of conditioning contraction intensities, even if contractions are performed at similar TTI. If no effect of conditioning contraction intensity with similar TTI is observed, this would mean that TTI is an important factor to consider when examining PAP, that is, contraction duration may compensate for contraction intensity to induce a given PAP.

## 2 METHODS

### 2.1 Ethics approval

An informed consent was obtained from all participants following a detailed explanation of all experimental procedures and associated risks. The study was approved by the ethics committee of Nantes University (no. 08042021) and was conducted according to the *Declaration of Helsinki*.

### 2.2 Participants

Twelve healthy males (mean ± SD: 74.7 ± 4.7 kg; 180.7 ± 5.6 cm), aged between 18 and 25 years (21.5 ± 1.7 years), volunteered to participate in this study. None of the participants had injuries on the lower limb. Before testing, participants were asked not to perform any strenuous exercise for at least 48 h. They were also asked to refrain from consuming caffeine, known to alter the magnitude of PAP, in the 24 h preceding the experiment (MacIntosh & Gardiner, 1987).

### 2.3 Experimental design and protocol

**FIGURE 1:**
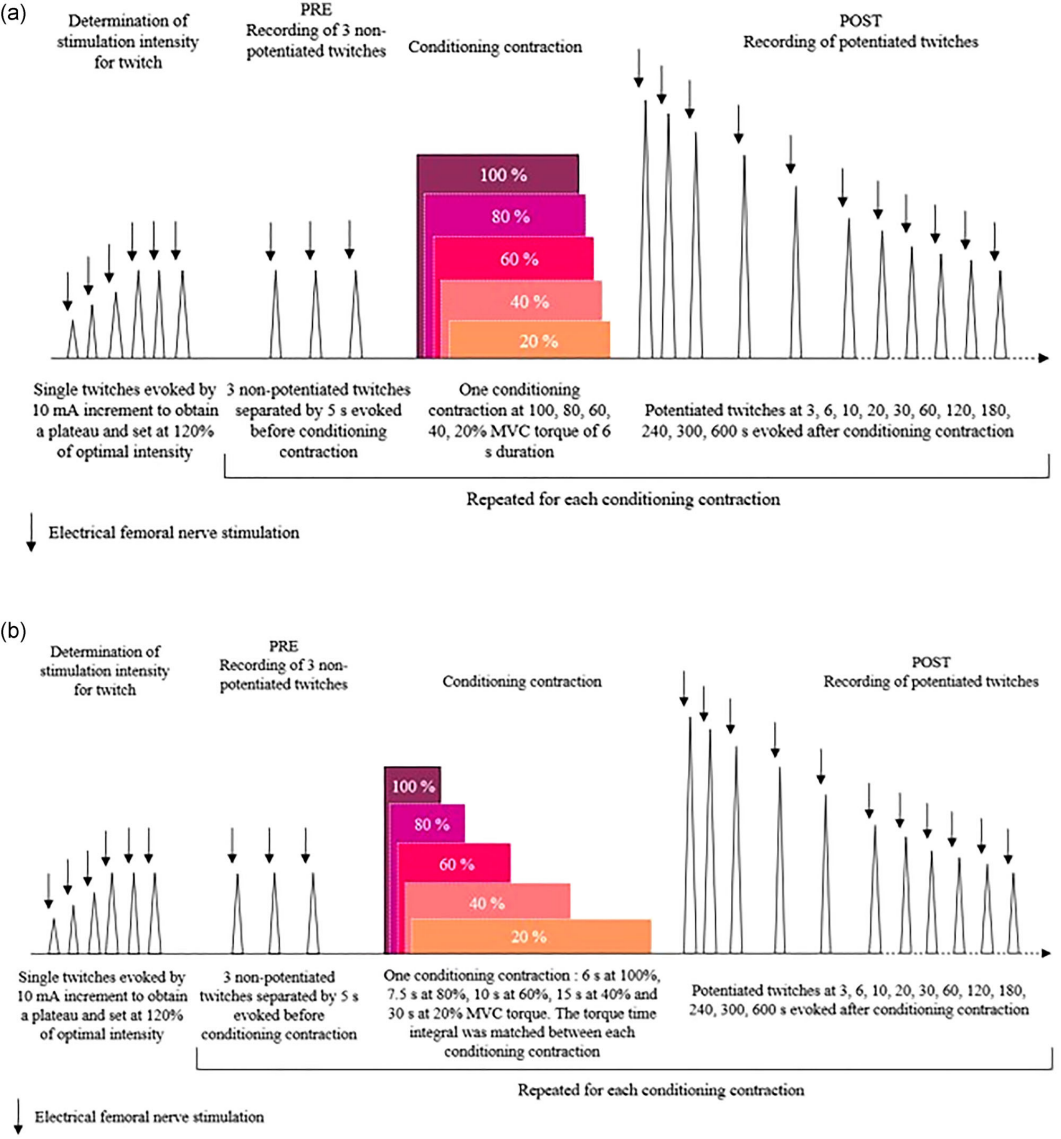
Overview of the experimental protocol for the first (a) and the second (b) experimental session. Experimental sessions consisted of evoking single twitches before (PRE, three twitches) and after (POST, 11 twitches) each conditioning contraction. In the first experimental session (session with different torque–time integral), one conditioning contraction of 6‐s duration was performed at 100, 80, 60, 40 and 20% of maximal voluntary contraction (MVC). In the second experimental session (session with similar torque–time integral), one conditioning contraction was also performed at 100, 80, 60, 40 and 20% of MVC. However, the duration of each conditioning contraction was adjusted so that the same torque–time integral was observed among the conditioning contractions. Therefore, the duration of each conditioning contraction was set up with respect to its intensity, that is, 6 s at 100%, 7.5 s at 80%, 10 s at 60%, 15 s at 40%, and 30 s at 20% MVC.

Participants were invited to participate in three laboratory sessions (one familiarization session and two experimental sessions), separated by at least 24 h. For each participant, all laboratory sessions were performed at the same time of day. All data were collected on the right leg. The familiarization session was used to accustom participants with isometric contractions and electrical nerve stimulation. During this session, participants performed five voluntary isometric contractions (100, 80, 60, 40 and 20% MVC) of the quadriceps. In the first experimental session (i.e., ‘session with different TTI’), transcutaneous femoral nerve stimulation was applied to evoke single twitches on the relaxed knee extensors at different time points before and after the voluntary isometric conditioning contractions. A series of three twitches, separated by 5 s, was evoked before each conditioning contraction. Single twitches were also evoked 3, 6, 10, 20, 30, 60, 120, 180, 240, 300 and 600 s after each conditioning contraction (Figure 1). A 6‐s conditioning contraction was performed at five intensity levels (100, 80, 60, 40 and 20% MVC). We chose this contraction duration on the basis of the study by Baudry and Duchateau (2007), which suggested that a 6‐s MVC may optimize PAP and avoid the fatigue that may coexist with PAP. In the second experimental session (i.e., ‘session with similar TTI’), a similar stimulation protocol was used. Conditioning contractions at the same five intensity levels were performed but the duration of each of them was adjusted to obtain a similar TTI between conditioning contractions (6 s at 100% MVC, 7.5 s at 80% MVC, 10 s at 60% MVC, 15 s at 40% MVC and 30 s at 20% MVC). In both experimental sessions, no warm‐up was performed to avoid any PAP on the twitches preceding the first conditioning contraction. Each experimental session started with the conditioning contraction at 100% MVC. The torque level reached during this contraction was used to determine the relative torque levels for the other conditioning contractions. The order of the submaximal conditioning contractions was then randomized. A new conditioning contraction started when the peak twitch torque returned to its mean initial value (±5%). Therefore, if the 10‐min recovery allocated after each conditioning contraction was not sufficient to return the twitch torque to its initial value, the resting period was prolonged. During each experimental session, on‐line visual torque feedback and a target line for submaximal contractions were provided on a computer screen to the participants.

### 2.4 Mechanical recordings

The torque signal was recorded to measure (i) the peak torques during twitches associated with femoral nerve stimulation and (ii) the torque associated with the conditioning contractions of the knee extensors. All knee extensor torque assessments were made on the right leg using a Biodex Dynamometer (Biodex 3, Shirley, NY, USA). Participants were seated on the dynamometer chair and were securely stabilized by using crossover shoulder harnesses to avoid the contribution of other muscle groups. Knee and hip joints were constantly fixed at 90° (0°: knee extended) and 100° (0°: hip extended), respectively, during all experimental sessions. The axis of the dynamometer was aligned with the anatomical knee flexion–extension axis and the lever‐arm was attached 3 cm above the lateral malleolus by using a strap. Torque signal was stored (sampling frequency 5 kHz) for recording with a software (BIOPAC System Inc., Goleta, CA, USA).

### 2.5 Femoral nerve stimulation

Transcutaneous femoral nerve stimulation was used to evoke twitches at rest before and after each conditioning contraction. Rectangular pulses (1 ms, 400 V maximal voltage) were delivered by using a constant current stimulator (Digitimer DS7AH, Digitimer Ltd, Welwyn Garden City, UK). Femoral nerve was stimulated using a cathode electrode (diameter: 10 mm, Kendall Medi‐Trace™, Tyco Healthcare, Pointe‐Claire, Quebec, Canada) located in the femoral triangle below the inguinal ligament. The anode electrode was a large electrode (89 × 50 mm, Chattanooga Medical Supply Inc., Chattanooga, TN, USA) located in the gluteal fold. Before the beginning of each experimental session, a recruitment curve of the resting single twitch was performed to determine the optimum stimulation intensity by progressively increasing current intensity from 10 mA until achieving maximal peak twitch torque. Maximal single peak twitch torque was considered to be reached when no improvement in peak twitch torque was observed between two stimulation intensities (Hucteau et al., 2020). A supra‐maximum stimulation intensity (120% of the last stimulation intensity used in the recruitment curve of the resting single twitch) was used during the experiment to guarantee the complete recruitment of motor units (Hucteau et al., 2020).

### 2.6 Data analysis

The 100% MVC torque was determined at the beginning of each experimental session to define the submaximal levels of voluntary contraction. It was considered as the highest peak torque value measured over three MVC trials. TTI was measured as the area under the torque–time curve of each conditioning contraction of the two experimental sessions. The peak torque of the twitches before and after all the conditioning contractions in both experimental sessions was measured. PAP was estimated by computing the ratio between the peak torque of each twitch evoked after the conditioning contraction and the mean of the three peak twitch torques before each conditioning contraction (i.e., PAP = (peak twitch torque after conditioning contraction/peak twitch torque before conditioning contraction) × 100). The variability between the peak twitch torques before conditioning contractions was lower than 5% in the session with different TTI (coefficient of variation (CV) = 3.28%) and with similar TTI (CV = 2.97%). All TTI values were calculated using a MATLAB program (MATLAB software, version R2017a, The MathWorks, Natick, MA, USA).

### 2.7 Statistical analysis

All statistical analyses were performed by using Statistica software for Windows (version 10.1, StatSoft, Tulsa, OK, USA). All data are presented as mean values ± standard deviation (SD). Significance was set at *P* < 0.05. The assumption of normality for all target variables was assessed using the Kolmogorov–Smirnov test. All variables followed a normal distribution (*K* = 0.1; *P *> 0.2). To control that twitch before each conditioning contraction was not potentiated or not altered by muscular fatigue, a two‐way ANOVA with repeated measures (session (‘different TTI’ and ‘similar TTI’) × intensity (20, 40, 60, 80 and 100% MVC)) was used on the twitch amplitude before conditioning contraction. A one‐way ANOVA was used to compare TTI values between conditioning contractions in each experimental session. A one‐way ANOVA was performed to compare TTI values between the same intensity levels of both experimental sessions. A three‐way ANOVA with repeated measures (session (‘different TTI’ and ‘similar TTI’) × intensity (20, 40, 60, 80 and 100% MVC) × time (3, 6, 10, 20, 30, 60, 120, 180, 240, 300 and 600 s)) was performed to compare the effect of TTI and intensity of conditioning contractions at different time points on the magnitude of PAP. A Tukey's HSD post hoc test was used when a significant effect or interaction between factors was observed. A coefficient of determination (*r* ^2^) was used to assess the strength of bivariate correlations (i) between the torque of conditioning contractions and PAP obtained 3 s after the conditioning contraction in sessions with and without similar TTI, and (ii) between the PAP values obtained 3 s after the conditioning contraction with and without similar TTI. With 12 participants, Pearson's correlation coefficient is considered significant (*P* < 0.05) when the *r* ^2^ value is ≥0.28.

## 3 RESULTS

### 3.1 Baseline measurements

No significant difference in the amplitudes of twitch preceding conditioning contractions was found between experimental sessions (effect of session: *P* = 0.176; *F* = 1.85; DF = 1) or conditioning contraction intensity (effect of intensity: *P* = 0.887; *F* = 0.28; DF = 4, interaction ‘session × intensity’: *P* = 0.448; *F* = 0.88; DF = 5). This indicates that twitches before each conditioning contraction have not been potentiated or altered by muscular fatigue.

No significant difference in MVC torque was observed between the two experimental sessions (‘session with different TTI’: 235.1 ± 31.7 N m; ‘session with similar TTI’: 235.7 ± 31.6 N m) (*P* = 0.9). Significant differences were found between all contraction intensities in the two experimental sessions (‘session with different TTI’: 80%: 189.8 ± 27.8 N m; 60%: 142 ± 17.4 N m; 40%: 95.4 ± 13.2 N m; 20%: 46.5 ± 7.9 N m; ‘session with similar TTI’: 80%: 188.3 ± 24.9 N m; 60%: 143.3 ± 21.6 N m; 40%: 98.5 ± 14.5 N m; 20%: 48.7 ± 6.6 N m) (all *P* < 0.001). Significant differences between all the five conditioning contractions were found when the TTI was unmatched between conditioning contractions (100%: 1388.8 ± 185.3 N m s; 80%: 1127.6 ± 160.6 N m s; 60%: 830 ± 89.9 N m s; 40%: 569.0 ± 70.6 N m s; 20%: 284.1 ± 35.6 N m s) (all *P* < 0.003). No significant difference between all the five conditioning contractions was found when the TTI was matched between conditioning contractions (100%: 1422.5 ± 190.2 N m s; 80%: 1389.1 ± 171.8 N m s; 60%: 1400.8 ± 185.2 N m s; 40%: 1406.2 ± 210.3 N m s; 20%: 1335.4 ± 160.7 N m s) (all *P* > 0.36). Comparing both experimental sessions, we did not observe a significant difference for TTI at 100% MVC (*P* = 0.67) while TTIs at 80, 60, 40 and 20% MVC were significantly greater in the session with similar TTI than in the session with different TTI (all *P* < 0.002).

### 3.2 Effect of conditioning contractions intensity on PAP

A significant interaction session × intensity × time was found for PAP (*P* = 0.035; *F* = 1.45; DF = 44).

### 3.3 PAP at different time points for each conditioning contraction intensity in the session with different TTI

**FIGURE 2:**
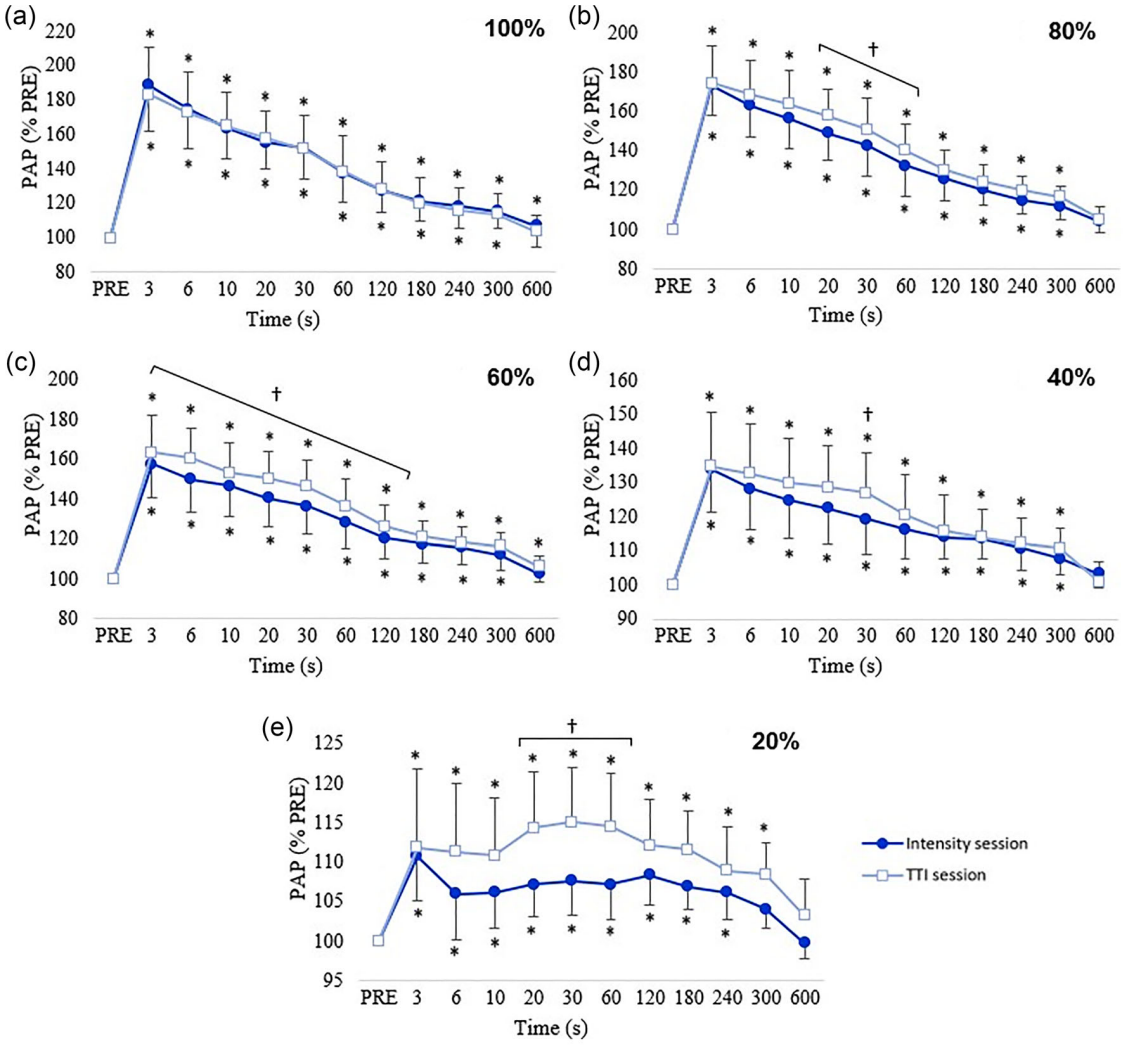
Change in post‐activation potentiation (PAP) at different time points after the conditioning contractions of the knee extensors with and without similar torque–time integral (TTI). (a, b, c, d, e) represent data for conditionning contractions of 100%, 80%, 60%, 40%, and 20% MVC, respectively. Session with different TTI (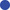), session with similar TTI (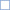). Mean values ± SD. *Significantly different from PRE (*P* < 0.05). ^†^Significantly different between sessions with and without similar TTI (*P* < 0.05).

After the conditioning contraction of 100% MVC, PAP was significantly observed at the time points from 3 to 600 s (all *P* < 0.002) (Figure 2a). After the conditioning contraction of 80, 60 and 40% MVC, PAP was significantly observed at the time points from 3 to 300 s (all *P* < 0.001) (Figures 2b–d). After the conditioning contraction of 20% MVC, PAP was significantly found at the time points from 3 s to 240 s (all *P* < 0.05) (Figure 2e).

**FIGURE 3:**
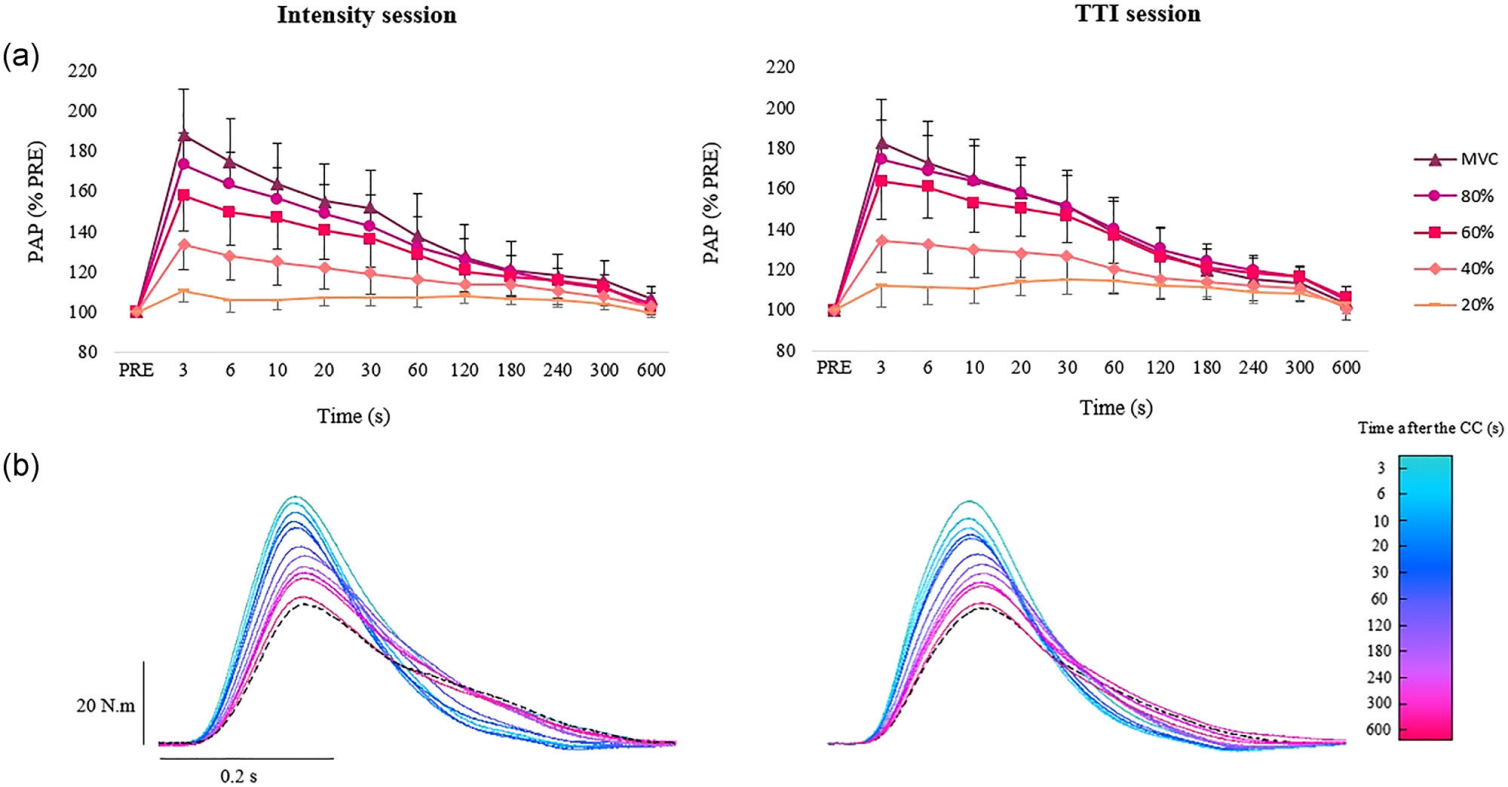
Amplitude of post‐activation potentiation (PAP) following conditioning contractions. (a) Change in PAP at different time points after the conditioning contractions of the knee extensors (3, 6, 10, 20, 30, 60, 120, 180, 240, 300, 600 s) in the sessions with similar torque–time integral (TTI) and with different TTI. 100% (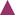), 80% (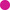), 60% (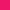), 40% (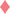), and 20% (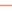) MVC. Mean values ± SD. A significant interaction session × intensity × time was found for PAP (*P* = 0.035; *F* = 1.45; DF = 44). For the sake of clarity, symbols for statistics significance are not presented on the figure. (b) Typical recordings of twitch torque from one subject before and after the conditioning 6‐s maximal voluntary contraction (MVC) at each time points in the session with similar and different TTI. The black dashed line represents the twitch torque recorded before the conditioning contraction. CC, conditioning contraction.

### 3.4 PAP between conditioning contractions intensities in the session with different TTI

PAP was significantly greater after conditioning contraction of 100% MVC than after conditioning contraction of (i) 80% MVC at the time points 3, 6, 10 and 30 s, (ii) 60% MVC at the time points from 3 to 120 s, (iii) 40% MVC at the time points from 3 to 300 s, and (iv) 20% MVC at the time points from 3 to 600 s (all 0.001 < *P* < 0.02) (Figure 3). PAP was significantly greater after conditioning contraction of 80% MVC than after conditioning contraction of (i) 60% MVC at the time points from 3 s to 60 s, (ii) 40% MVC at the time points from 3 to 180 s, and (iii) 20% MVC at the time points from 3 to 300 s (all 0.001 < *P* < 0.02) (Figure 3). PAP was significantly greater after conditioning contraction of 60% MVC than after conditioning contraction of (i) 40% MVC at the time points from 3 s to 60 s, and (ii) 20% MVC at the time points from 3 to 300 s (all *P* < 0.001) (Figure 3). PAP was significantly greater after conditioning contraction of 40% MVC than after conditioning contraction of (i) 20% MVC at the time points from 3 to 240 s (all 0.001 < *P* < 0.02) (Figure 3).

### 3.5 PAP at different time points for each conditioning contraction intensity in the session with different TTI

After the conditioning contraction of 100, 80, 40 and 20%, PAP was significantly observed at the time points from 3 s to 300 s (all *P* < 0.001) (Figure 2a, b, d, e). After the conditioning contraction of 60% MVC, PAP was significantly observed at the time points from 3 s to 600 s (all *P* < 0.03) (Figure 2c).

### 3.6 PAP between conditioning contractions intensities in the session with similar TTI

PAP was significantly greater after conditioning contraction of 100% MVC than after conditioning contraction of (i) 80% MVC at the 3‐s time points, (ii) 60% MVC at the time points from 3 to 20 s, (iii) 40% MVC at the time points from 3 to 180 s, and (iv) 20% MVC at the time points from 3 to 240 s (all 0.001 < *P* < 0.04) (Figure 3). PAP was significantly greater after conditioning contraction of 80% MVC than after conditioning contraction of (i) 60% MVC at the time points from 3 s to 20 s, (ii) 40% MVC at the time points from 3 to 240 s, and (iii) 20% MVC at the time points from 3 to 300 s (all *P* < 0.001) (Figure 3). PAP was significantly greater after conditioning contraction of 60% MVC than after conditioning contraction of (i) 40% MVC at the time points from 3 s to 300 s, and (ii) 20% MVC at the time points from 3 to 300 s (all 0.001 < *P* < 0.03) (Figure 3). PAP was significantly greater after conditioning contraction of 40% MVC than after conditioning contraction of (i) 20% MVC at the time points from 3 to 30 s (all *P* < 0.001) (Figure 3).

### 3.7 Comparison of PAP between sessions with similar versus different TTI

At each time point after the conditioning contraction of 100% MVC, PAP was not significantly different between sessions with similar TTI and with different TTI (all *P* > 0.85) (Figure 2a). After the 80% MVC conditioning contraction, PAP was significantly greater with similar TTI than with different TTI at the time points from 20 s to 60 s (all *P* < 0.02) (Figure 2b). After the 60% MVC conditioning contraction, PAP was significantly greater with similar TTI than with different TTI at the time points from 3 s to 120 s (all *P* < 0.001) (Figure 2c). After the 40% MVC conditioning contraction, PAP was significantly greater with similar TTI than with different TTI at the 30‐s time point (*P* = 0.031) (Figure 2d). After the 20% MVC conditioning contraction, PAP was significantly greater with similar TTI than with different TTI at the time points from 20 to 60 s (all *P* < 0.004) (Figure 2e).

### 3.8 Correlations between PAP and the intensity of conditioning contraction with and without similar TTI

**FIGURE 4:**
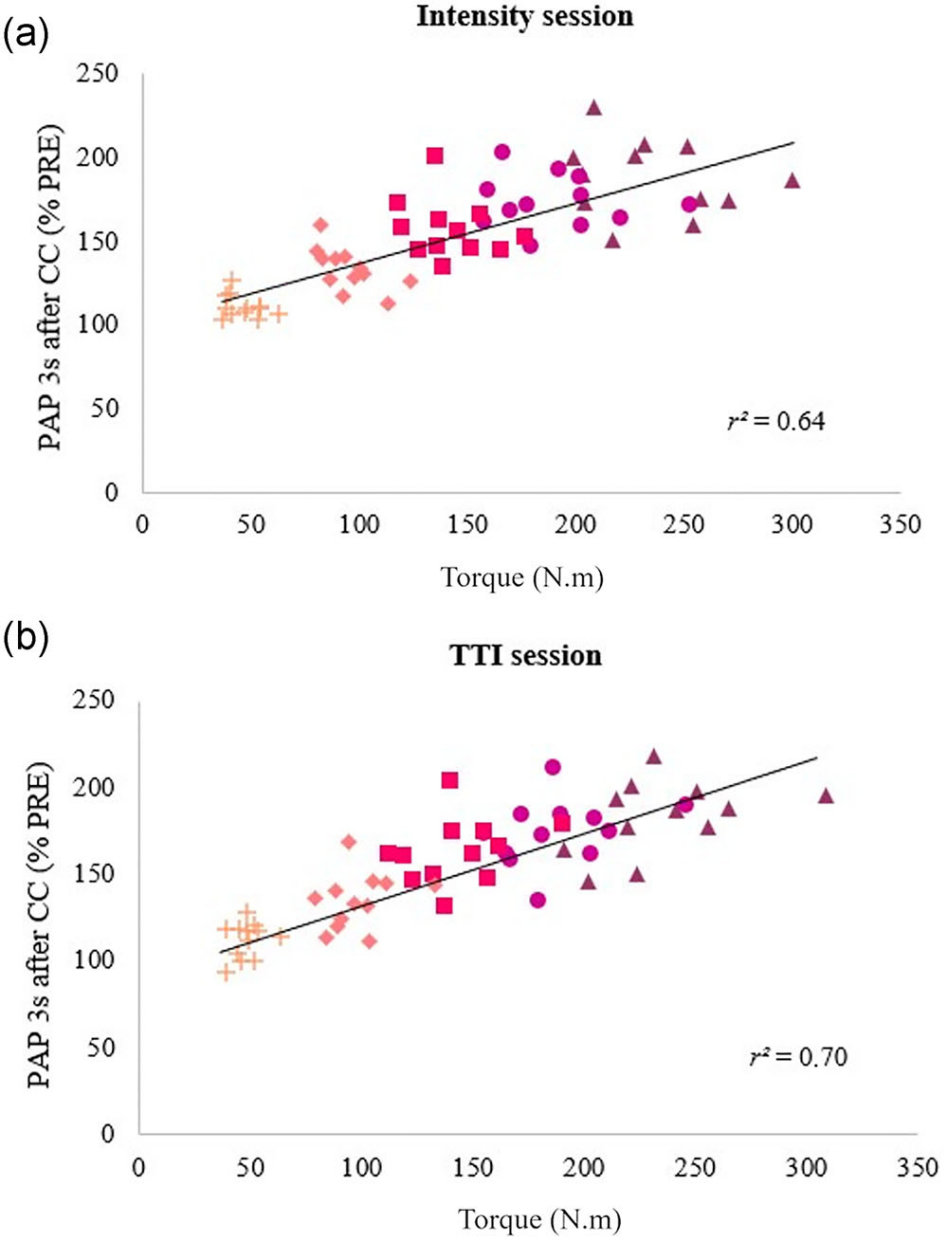
Correlations between post‐activation potentiation (PAP) at 3 s after the conditioning contractions and torque of the conditioning contractions in sessions without (a) and with (b) similar torque–time integral (TTI). 100% MVC (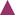), 80% MVC (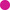), 60% MVC (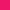), 40% MVC (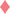), 20% MVC (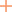). Each symbol (mean) represents one subject. CC, conditioning contraction.

**FIGURE 5:**
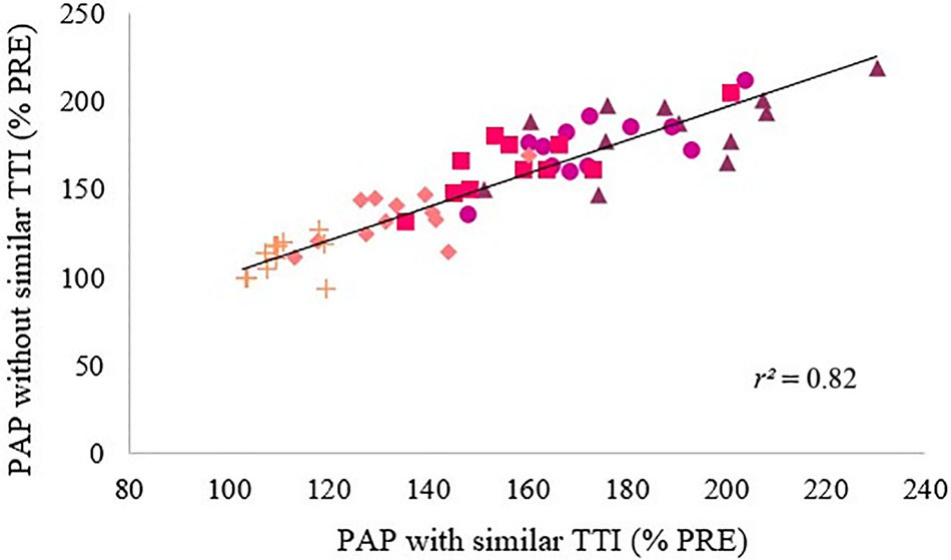
Correlation between post‐activation potentiation (PAP) at 3 s after conditioning contractions without and with similar torque–time integral (TTI). 100% MVC (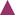), 80% MVC (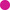), 60% MVC (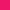), 40% MVC (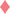), 20% MVC (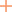). Each symbol (mean) represents one subject.

A positive correlation was found between TTI and PAP at 3 s after the conditioning contractions with different TTI (*r* ^2^ = 0.66; *P* < 0.001). Positive correlations were found between PAP at 3 s after the conditioning contractions and torque of the conditioning contractions with different TTI (*r* ^2^ = 0.64; *P* < 0.001) and with similar TTI (*r* ^2^ = 0.70; *P* < 0.001) (Figure 4). A positive correlation was found between PAP at 3 s after the conditioning contractions with similar and different TTI (*r* ^2^ = 0.82; *P* < 0.001) (Figure 5).

## 4 DISCUSSION

In this study, we compared the PAP magnitude between different conditioning contraction intensities performed with and without similar TTI in the knee extensors. First, our results showed that PAP magnitude is strongly influenced by conditioning contraction intensity, whether the conditioning contraction intensities are performed with or without similar TTI. Second, the relationships between PAP and conditioning contraction intensity were quite similar, whether contractions were performed with or without a similar TTI. Third, there was a strong relationship between PAP amplitudes following conditioning contractions with and without similar TTI. Together, these results suggest that TTI has a minor influence on PAP in the knee extensors. In other words, a modulation of conditioning contraction intensity may not be compensated by a proportional modulation of conditioning contraction duration to obtain a given PAP magnitude.

### 4.1 Contraction intensity and post‐activation potentiation

In agreement with previous work (Fukutani et al., 2014; Mettler & Griffin, 2012; Sasaki et al., 2012; Vandervoort et al., 1983), our results confirmed the strong influence of muscle contraction intensity on PAP magnitude. We found that PAP increases according to the increase of conditioning contraction intensity from 20 to 100% MVC, regardless of the time points. We also observed a strong relationship between the PAP assessed 3 s after the conditioning contraction and the absolute knee extensors torque in both sessions with or without similar TTI. The relationship between PAP and conditioning contraction intensity was jointly attributed to the greater phosphorylation of regulatory myosin light chains in fast‐twitch fibres compared to slow‐twitch fibres and the greater activation of fast‐twitch fibres at high than low levels of muscle contraction (Brown & Loeb, 1998; Grange et al., 1993, 1998; Hamada et al., 2003; Houston et al., 1985; Moore & Stull, 1984; Sweeney et al., 1993; Vandervoort et al., 1983). Indeed, according to the Henneman principle (Henneman et al., 1965), in physiological circumstances small motor units, mostly activating slow‐twitch fibres, are always recruited first. When more force is required, larger motor units, mostly activating fast‐twitch fibres, are recruited additionally. Furthermore, our results are in accordance with several studies showing that the further the PAP is assessed from the conditioning contraction, the smaller the effect of the conditioning contraction intensity on PAP (Folland et al., 2008; Jubeau et al., 2010; Mettler & Griffin, 2012; Pääsuke et al., 2007; Requena et al., 2008). In our study, we found that this occurred for the conditioning contractions from 40 to 100% MVC. Nevertheless, the amplitude of PAP was higher from 20 to 60 s after 20% MVC, which could be explained by a higher sensitivity of Ca^2+^ several seconds after low‐intensity exercise (Boullosa et al., 2018).

### 4.2 Torque–time integral and post‐activation potentiation

The association between PAP and conditioning contraction intensity was only slightly different between sessions with and without similar TTI. Indeed, over the 44 PAP obtained during submaximal efforts (four submaximal contraction intensities × 11 time intervals), only 14 were significantly greater in the session with similar TTI than in the session with different TTI, including four after low levels of effort (20 and 40% MVC) and 10 after moderate and high levels of effort (60 and 80% MVC). This difference in time course between both sessions could be attributed to the longer contraction duration performed in the session with matched TTI. It has been shown after prolonged exercise at submaximal intensities that the sensitivity to Ca^2+^ was maintained (Hvid et al., 2013), which may contribute to maintaining a higher PAP over the time after a longer duration of submaximal conditioning contraction. We also found that the relationship between PAP and conditioning contraction intensity was quite similar between the sessions with (*r* ^2^ = 0.70) and without (*r* ^2^ = 0.64) similar TTI. Since similar TTI across the different levels of conditioning contraction did not reduce the association between PAP and conditioning contraction intensity compared to different TTI, this means that a modulation of contraction intensity cannot be compensated by a proportional modulation of contraction duration. Moreover, we observed a high level of covariation (*r* ^2^ = 0.82) between the PAP induced by conditioning contractions with similar TTI and the PAP induced by conditioning contractions with different TTI. Taken together, our results show that for conditioning contraction ≥6 s, PAP magnitude in the knee extensors is mainly sensitive to conditioning contraction intensity and poorly sensitive to TTI and duration of the conditioning contraction.

Our data are not in agreement with those of Mettler and Griffin (2012) in which no statistical change in PAP was observed between different conditioning contraction intensities with similar TTI (20 s at 25%, 10 s at 50%, and 5 s at 100% MVC). This previous report suggested that a modulation of contraction duration may inversely compensate for a modulation of contraction intensity. Unlike the results of Mettler and Griffin (2012), we demonstrated a low effect of contraction duration since the contraction intensity effect has not been compensated by contraction duration. We found that whether there was similar TTI (6 s at 100%, 7.5 s at 80%, 10 s at 60%, 15 s at 40%, and 30 s at 20%) or not (6 s for all conditioning contractions intensities), the PAP was significantly different between conditioning contraction intensities. The conflicting results between our study and the one of Mettler and Griffin (2012) can be explained by the difference in the muscle group tested since PAP is muscle‐dependent (Fukutani et al., 2014). While we assessed PAP in the quadriceps, Mettler and Griffin (2012) assessed PAP in the adductor pollicis muscle. The adductor pollicis muscle is mostly composed of slow‐twitch fibres (about 80%) and the quadriceps have about as many slow‐twitch fibres as fast‐twitch fibres (Vikne et al., 2012). Since slow‐twitch fibres are less sensitive to PAP than fast‐twitch fibres (Brown & Loeb, 1998; Grange et al., 1993; Hamada et al., 2003; Houston et al., 1985; Moore & Stull, 1984; Vandervoort et al., 1983), the difference in fibre composition between both muscles may explain the divergent results between studies.

It is important to take into consideration the potential coexistence of fatigue and PAP following conditioning contractions (Rassier & MacIntosh, 2000). Indeed, fatigue leads to a decrease in contractile force, which means that PAP and fatigue processes could be simultaneous and underestimate the magnitude of PAP. It has been shown that a longer duration of conditioning contraction induces lower PAP due to the fatigue (Vandervoort et al., 1983). Thus, it is reasonable to assume that if muscle fatigue occurs with higher conditioning contraction duration such as 30 s in 20% MVC and 15 s at 40% MVC in our study, this may have altered the PAP. Indeed, PAP does not occur for three participants (out of 12) during 20% MVC of the FTI session. However, Mettler & Griffin (2012) showed that repeated 5‐s 50 and 25% MVC was not correlated with the fatigue index. In addition, the same authors demonstrated that the optimal duration of conditioning contraction decreased as the intensity of conditioning contraction increased to obtain the maximal potentiation, which provides evidence that the effect of fatigue may have been minimal in our study.

Finally, there was no difference between the amplitude of twitches preceding conditioning contractions. This indicates that twitches before conditioning contractions have not been potentiated or not altered by muscle fatigue.

### 4.3 Conclusion

The findings of this study demonstrate that quadriceps conditioning contractions with and without similar TTI have close effects on PAP, even when the conditioning contraction intensities differ. In addition, the magnitudes of PAP at 3 s after conditioning contractions with and without similar TTI are highly correlated. However, we can notice that the time course of PAP can slightly vary, depending on the conditioning contraction intensity. Overall, this suggests that to maximize PAP magnitude in the quadriceps with an isometric effort of at least 6 s, it is important to primarily control the intensity of the conditioning contractions.

## AUTHOR CONTRIBUTIONS

Marc Jubeau and Thomas Cattagni conceived and designed the research. Pauline Eon conducted experiments and extracted results. Pauline Eon, Thomas Cattagni, and Marc Jubeau analyzed the data. Pauline Eon, Marc Jubeau, and Thomas Cattagni wrote the manuscript. All authors have read and approved the final version of this manuscript and agree to be accountable for all aspects of the work in ensuring that questions related to the accuracy or integrity of any part of the work are appropriately investigated and resolved. All persons designated as authors qualify for authorship, and all those who qualify for authorship are listed.

## CONFLICT OF INTEREST

The authors declare no conflicts of interest.

### FUNDING INFORMATION

No funding was received for this work.

## Data Availability

The results of the study are presented clearly, honestly, and without fabrication, falsification, or inappropriate data manipulation. All data are available upon request to the authors.
